# Role of Shame and Concealment in Patient-Provider Communication Among Individuals With Low Health Literacy: Protocol for a Scoping Review

**DOI:** 10.2196/83451

**Published:** 2026-01-30

**Authors:** Tsuyoshi Okuhara, Hiroko Okada, Rie Yokota

**Affiliations:** 1 Department of Health Communication, School of Public Health, The University of Tokyo, 7-3-1 Hongo, Bunkyo-ku, Tokyo, Japan, 81 1-3-5800-6549; 2 Department of Medical Communication, School of Pharmacy and Pharmaceutical Sciences, Hoshi University, Tokyo, Japan

**Keywords:** health literacy, low health literacy, communication, professional-patient relations, shame, concealment, qualitative research, scoping review

## Abstract

**Background:**

Health literacy, defined as the ability to obtain, understand, evaluate, and use health information, influences health behaviors and outcomes. Low health literacy (LHL) is associated with misunderstandings of treatment instructions, poor adherence, and inadequate preventive behaviors, all of which contribute to health disparities. Although universal precautions such as plain language and the teach-back method are recommended, recent studies indicate that these measures alone cannot fully address the challenges faced by patients with LHL. Previous qualitative studies have examined psychosocial processes through which shame and concealment shape patient-provider communication; however, these findings remain fragmented across settings and disciplines, and no scoping or systematic review has yet synthesized this evidence.

**Objective:**

This review aims to map how patients with LHL experience shame and concealment, how health care providers’ behaviors and communication influence these experiences, and how these processes affect patient-provider communication and care. It also seeks to identify strategies to reduce the impact of shame and concealment in clinical practice.

**Methods:**

This review will be conducted and reported in accordance with the PRISMA-ScR (Preferred Reporting Items for Systematic Reviews and Meta-Analyses extension for Scoping Reviews) guidelines. PubMed, MEDLINE, CINAHL, PsycInfo, Web of Science, and Academic Search Complete will be searched using terms related to health literacy, patient-provider communication, and qualitative research. Qualitative and mixed methods studies with qualitative findings will be included, and quantitative-only studies will be excluded. The participants may include patients, health care providers, or both. Data extraction will include specific manifestations of shame (eg, embarrassment, fear of judgment, and self-blame), concealment behaviors (eg, silence, avoidance, and impression management), provider communication behaviors (eg, time pressure, use of jargon, dismissiveness, and validation), and key findings. The accuracy of the data will be verified by multiple reviewers. Data will be synthesized using thematic synthesis, with the findings presented in tables; narrative synthesis; and a conceptual model depicting the interactions among shame, concealment, and provider communication. The findings will describe how shame and concealment are conceptualized, how they shape communication and care, and strategies suggested to reduce their effects. A conceptual diagram will illustrate these dynamics.

**Results:**

This study was funded in April 2025. Database searching is scheduled for October 2025, with study selection and data extraction planned for November 2025. As of September 2025, no data extraction has been completed. Data synthesis is expected to be finalized by December 2025, and results are planned for publication between June and August 2026.

**Conclusions:**

This will be the first scoping review to systematically map the roles of shame and concealment in health care communication among patients with LHL. Synthesizing qualitative evidence will provide insights into relational dynamics, inform professional education and training, and guide organizational- and policy-level strategies to promote equitable and patient-centered health care communication.

## Introduction

Health literacy is defined as the ability to obtain, understand, evaluate, and use health information and has been recognized as a crucial factor influencing health behaviors and outcomes [1-3]. Low health literacy (LHL) has been associated with problems such as misunderstanding medication instructions, poor treatment adherence, and inadequate engagement in preventive behaviors [4-6] and has been identified as a risk factor contributing to health disparities and failures in chronic disease management [7-9]. Recent systematic and scoping reviews continue to demonstrate that health literacy is strongly associated with self-management, medication adherence, quality of life, and clinical outcomes across a wide range of chronic conditions [4-6,10]. To address these challenges, “universal precautions” in health literacy have been recommended, including the use of plain language and the teach-back method, to ensure understanding regardless of patients’ literacy levels [11].

However, studies have highlighted the limitations of explaining the difficulties of patients with LHL solely in terms of “deficits in information-processing skills.” Particular attention has been paid to the psychosocial mechanisms underlying shame and concealment. Parikh et al [12] reported in their seminal study that 42.6% of patients had inadequate or marginal health literacy, nearly 40% of those acknowledging reading difficulties felt ashamed, 19% never disclosed their difficulties to anyone, and over 60% avoided seeking help. In their subsequent study, 47.8% of patients with the lowest literacy level reported shame compared to only 6.5% at higher levels. Although most patients (83.7%) agreed that documenting literacy difficulties in medical records was useful, those with lower literacy levels were significantly more likely to experience embarrassment, which hindered open communication with providers [13]. These findings have since been interpreted through broader stigma frameworks, which conceptualize shame not merely as an individual emotion but as a socially produced response to anticipated devaluation and power asymmetries in health care interactions [14-16].

Qualitative studies have confirmed that shame and concealment profoundly affect health care communication. Easton et al [17] described how patients with low literacy and fear might be perceived as ignorant, avoid asking questions, and leave consultations without sufficient understanding. Other qualitative studies have also revealed that several patients feel inferior when interacting with those who possess medical expertise, leading them to hesitate to ask questions or express disagreement [18,19]. Recent studies have highlighted the broader social and systemic contexts in which these experiences occur. For example, Stormacq et al [20] observed that socioeconomically disadvantaged adults experience health literacy as an “obstacle course,” where financial insecurity heightens their need for information but simultaneously restricts access to physicians, leading many to depend on less reliable sources such as the internet or relatives. In this context, difficulties in understanding or evaluating health information are often accompanied by feelings of shame and a tendency toward concealment, which discourage patients from asking questions or disclosing their struggles [20].

In parallel, a growing body of qualitative literature has examined how health care providers’ behaviors and communication styles may unintentionally reinforce these psychosocial dynamics. For example, Shaw et al [21] reported that patients with LHL often felt reluctant to ask questions due to physicians’ short consultation times, insufficient information provided, and complex terminology used, which ultimately resulted in patient silence or concealment. Similarly, Jensen et al [22] showed that patients with lower literacy were more likely to feel that their voices were not respected by health care providers and that health care providers did not listen adequately, which contributed to lower satisfaction and inhibited patients’ willingness to speak up. Recent studies in primary care, physiotherapy, cardiovascular prevention, and palliative care have further demonstrated that providers often struggle to accurately identify LHL and consistently apply communication strategies tailored to patients’ needs despite strong normative support for health literacy–friendly practices [23-26].

Taken together, these findings indicate that shame and concealment are not isolated individual reactions but relational processes shaped by social context, stigma, and the communicative practices of health care professionals. If left unaddressed, these processes reinforce silence, misunderstanding, and inequities in care. Nevertheless, despite a growing body of qualitative literature, existing research remains highly fragmented across clinical domains (eg, primary care, chronic disease management, rehabilitation, and palliative care), populations (eg, older adults, socioeconomically disadvantaged groups, and culturally diverse communities), and conceptual frameworks. Some studies foreground shame without explicitly examining concealment behaviors [17,18], others focus on provider communication without directly theorizing emotional processes [23,26], and still others examine stigma at a general level without anchoring it to health literacy–specific interactions [16].

To date, no scoping or systematic review has synthesized this heterogeneous qualitative evidence to clarify how shame and concealment operate as dynamic psychosocial mechanisms within health care communication for patients with LHL. As a result, it remains unclear how these processes unfold across settings, how they are shaped by provider communication styles, and which strategies are most promising for reducing their harmful effects.

This scoping review will focus exclusively on the findings of qualitative and mixed methods studies. This is because phenomena such as shame and concealment are deeply rooted in patients’ subjective experiences and emotions and cannot be adequately captured through quantitative measures alone. Importantly, the rationale for excluding quantitative-only studies is not based on a denial of the value of quantitative emotion research. Rather, this review specifically aims to elucidate how shame and concealment are experienced, negotiated, and managed within patient-provider interactions as social and relational processes. These dynamic meaning-making processes are best accessed through qualitative data such as narratives, interviews, and observational accounts. Recent conceptual work on epistemic humility and epistemic justice further underscores the importance of examining how patients’ voices are validated or silenced in clinical interactions, particularly in contexts marked by informational and power asymmetries [27,28]. By synthesizing qualitative research, this review seeks to comprehensively illustrate how patients experience shame, how concealment behaviors arise, and how these behaviors are shaped by social and cultural factors as well as interactions with health care providers.

Existing studies on this topic remain heterogeneous and fragmented, encompassing diverse populations, including adults, older people, low-income groups, and those from various cultural backgrounds. Therefore, this study aims to map the range of relevant research and organize the conceptual and theoretical frameworks. Therefore, a scoping review approach is more appropriate than a systematic review approach.

The following research questions will guide this review:

- How do patients with LHL experience shame and concealment?
- How do health care providers’ behaviors (eg, impression-based judgments and labeling) influence patients’ experiences of shame and concealment?
- How do the processes of shame and concealment affect patient-provider communication and quality of care?
- What strategies have been proposed to mitigate the effects of shame and concealment in clinical practice?

## Methods

### Overview

This scoping review will be conducted following the PRISMA-ScR (Preferred Reporting Items for Systematic Reviews and Meta-Analyses extension for Scoping Reviews) guidelines [29]. A completed PRISMA-ScR checklist will be provided as a supplementary file upon manuscript submission. The methodological framework is guided by the seminal work by Arksey and O’Malley [30] on scoping studies, further refined by the work by Levac et al [31] and updated with methodological guidance for conducting scoping reviews [32]. We plan to initiate the database search in October 2025, complete study selection and data extraction by November 2025, and finalize data synthesis by December 2025. In accordance with the primary objective of this scoping review—to map the range, nature, and conceptualization of qualitative evidence rather than evaluate intervention effectiveness or methodological rigor—we will not conduct a formal critical appraisal of individual studies. This approach is consistent with established scoping review methodology and PRISMA-ScR recommendations [29].

### Literature Search

We will search the following databases: PubMed, MEDLINE, CINAHL, PsycInfo, Web of Science, and Academic Search Complete. The search strategy will combine MeSH (Medical Subject Headings) and free-text terms related to health literacy and patient-provider communication. Textbox 1 shows the combination of keywords that will be used in our search. We will not filter our searches based on the year of publication. The reference lists of eligible articles and relevant reviews will also be screened for additional information.

Textbox 1. **Search terms.**

**PubMed**

- (doctor*[Title/Abstract] OR physician*[Title/Abstract] OR nurs*[Title/Abstract] OR clinician*[Title/Abstract] OR professional*[Title/Abstract] OR provider*[Title/Abstract] OR expert*[Title/Abstract] OR patient*[Title/Abstract] OR adult*[Title/Abstract] OR “lay person”[Title/Abstract] OR laypeople[Title/Abstract] OR Physicians[MeSH Terms] OR Patients[MeSH Terms] OR Adult[MeSH Terms] OR “Health Personnel”[MeSH Terms]) AND (“health literacy”[Title/Abstract] OR literacy[Title/Abstract] OR “Health Literacy”[MeSH Terms]) AND (qualitative[Title/Abstract] OR interview*[Title/Abstract] OR “focus group”[Title/Abstract] OR “Qualitative Research”[MeSH Terms])

**MEDLINE**

- XB(“doctor*” OR “physician*” OR nurs*[Title/Abstract] OR “clinician*” OR “professional*” OR “provider*” OR “expert*” OR “patient*” OR “adult*” OR “lay person” OR “laypeople”) AND
- XB(“health literacy” OR literacy) AND XB(“qualitative” OR “interview*” OR “focus group”)

**CINAHL**

- XB(“doctor*” OR “physician*” OR nurs*[Title/Abstract] OR “clinician*” OR “professional*” OR “provider*” OR “expert*” OR “patient*” OR “adult*” OR “lay person” OR “laypeople”) AND XB(“health literacy” OR literacy) AND XB(“qualitative” OR “interview*” OR "focus group)

**PsycInfo**

- XB(“doctor*” OR “physician*” OR nurs*[Title/Abstract] OR “clinician*” OR “professional*” OR “provider*” OR “expert*” OR “patient*” OR “adult*” OR “lay person” OR “laypeople”) AND XB(“health literacy” OR literacy) AND XB(“qualitative” OR “interview*” OR “focus group”)

**Academic Search Complete**

- XB(“doctor*” OR “physician*” OR nurs*[Title/Abstract] OR “clinician*” OR “professional*” OR “provider*” OR “expert*” OR “patient*” OR “adult*” OR “lay person” OR “laypeople”) AND XB(“health literacy” OR literacy) AND XB(“qualitative” OR “interview*” OR “focus group”)

**Web of Science**

- TS=(“doctor*” OR “physician*” OR nurs*[Title/Abstract] OR “clinician*” OR “professional*” OR “provider*” OR “expert*” OR “patient*” OR “adult*” OR “lay person” OR “laypeople”) AND TS=(“health literacy” OR literacy) AND TS=(“qualitative” OR “interview*” OR “focus group”)

### Eligibility Criteria

Textbox 2 shows the inclusion and exclusion criteria for this study.

Textbox 2. **Inclusion and exclusion criteria.**

**Inclusion criteria**

- Studies involving health care providers, patients, or both
- Studies focusing on patients with low health literacy and their experiences of shame, concealment, or related psychosocial processes in health care contexts
- Studies examining the role of health care providers’ communication or behavior in relation to patient shame and concealment
- Qualitative or mixed methods studies including qualitative findings relevant to shame or concealment
- Studies involving participants of any age, sex, ethnicity, or country
- Peer-reviewed articles and gray literature (eg, theses and conference proceedings) providing sufficient methodological details
- Papers written in English
- Studies published in any year

**Exclusion criteria**

- Quantitative studies that only measure health literacy levels
- Quantitative-only studies that do not include qualitative data capable of supporting thematic synthesis of shame, concealment, or interactional processes
- Studies exclusively focusing on general stigma unrelated to health literacy
- Unavailable full texts or articles not written in English

### Study Selection

We will use the Rayyan software (Qatar Computing Research Institute) [33] to manage and screen the citations. Duplicates will be removed automatically. Two reviewers (TO and HO) will independently screen the titles and abstracts against the inclusion criteria. Disagreements will be resolved through discussion, and if a consensus cannot be reached, a third reviewer (RY) will be consulted. The same process will be applied to the full-text screening. The selection process will be documented using a PRISMA-ScR flow diagram.

### Data Extraction

One reviewer (TO) will extract the data using a standardized form developed in Microsoft Excel. The following data will be extracted:

- Bibliographic details (author, year, country, and journal)
- Study design and methodology
- Participant characteristics (sample size, demographics, and clinical setting)
- Specific emotional expressions of shame (eg, embarrassment, humiliation, and fear of negative evaluation), concealment behaviors (eg, silence, pretending to understand, avoidance of questions, and impression management)
- Specific provider communication and behavioral features (eg, consultation time constraints, use of medical jargon, dismissive responses, validation, and teach-back method use)
- Key findings (themes, categories, and representative quotations)
- Implications for clinical practice, education, and policy

To ensure accuracy, the second (HO) and third (RY) reviewers will verify the extracted data for omissions or errors.

### Data Synthesis

The initial data synthesis will be conducted by the first reviewer (TO) using thematic synthesis [34]. This approach, endorsed by The Cochrane Collaboration as a rigorous strategy for integrating qualitative evidence [35], involves several stages. In the first stage, TO, who has a background in qualitative methodology and extensive experience in conducting qualitative research, will perform open, line-by-line coding of the findings and quotations presented in the results sections of the included articles. In the second stage, both the first (TO) and second (HO) reviewers will independently cluster the codes that emerged from the initial coding into descriptive themes that will be data driven. Any discrepancies will be resolved through discussion with a third reviewer (RY). In the third stage, TO will generate analytical themes by interpreting and extending the descriptive themes from the previous step. These analytical themes will aim to provide insights beyond the findings of individual studies. The development of these themes will be discussed collaboratively among TO, HO, and RY, and all codes and themes will be created inductively through this iterative synthesis process. In addition, study characteristics (eg, setting, population, country, and clinical context) and intervention-related information will be summarized using descriptive numerical analysis and structured tables. These multiple layers of synthesis will be integrated to construct a conceptual model that visually represents the relational pathways linking health literacy, shame, concealment, provider behaviors, and communication outcomes.

### Dissemination

We will present the findings in both a summary table and conceptual diagram and elaborate on them through descriptive and narrative synthesis. A conceptual model will be constructed to depict the interplay between patients’ experiences of shame and concealment and the behaviors and communication of health care providers. Following completion of data synthesis, the findings will be submitted to a peer-reviewed journal and disseminated at international conferences.

## Results

This study was funded in April 2025. Database searching is scheduled for October 2025, with study selection and data extraction planned for November 2025. As of September 3, 2025, no data extraction has been completed. Data synthesis is expected to be finalized by December 2025, and results are planned for publication between June and August 2026.

## Discussion

### Anticipated Findings

This scoping review will be the first to systematically map how shame and concealment are experienced by patients with LHL and how health care providers’ behaviors may exacerbate or mitigate these experiences. By synthesizing evidence from qualitative and mixed methods studies with qualitative findings, our review will provide a more comprehensive understanding of this psychosocial phenomenon.

Shame and concealment are not isolated experiences but relational processes shaped by clinical encounters and broader social contexts. Previous studies have shown that patients with LHL often hide their reading or comprehension difficulties out of fear of being judged, which prevents them from accessing the necessary support [18,19]. Such concealment can act as a coping mechanism, protecting patients from embarrassment while simultaneously restricting their ability to obtain information and engage fully in care [13]. Our review will highlight how these dynamics contribute to health inequities, particularly among populations already disadvantaged by socioeconomic or cultural factors.

These findings will have implications for both clinical practice and professional training. Health care providers may unintentionally reinforce shame through hurried communication, the use of jargon, or assumptions about patients’ abilities [21]. For example, patients often report that rush consultations and lack of plain language increase their reluctance to ask questions [22]. By mapping the available evidence, this review will identify strategies that can reduce these risks, such as adopting plain language, using the teach-back method, and creating safe spaces for patient questions.

### Limitations

This study may have some limitations. First, because we will restrict our inclusion to studies published in English, relevant evidence reported in other languages may be overlooked. This limitation is particularly important given that health literacy and the associated experiences of shame and concealment are strongly influenced by cultural and linguistic contexts. Second, although we will attempt to identify gray literature, some relevant reports, theses, or conference proceedings may not be retrieved because of limited indexing. Third, in accordance with the objectives of this scoping review, we will not quantitatively evaluate the effectiveness of the interventions. Consequently, our findings will not provide pooled estimates or definitive conclusions regarding intervention outcomes. Despite these limitations, the scoping review approach is well suited to our aims as it enables us to capture a broad and heterogeneous body of evidence, clarify conceptual boundaries, and identify important gaps in future research.

### Conclusions

To the best of our knowledge, this will be the first scoping review to systematically map how shame and concealment are experienced by patients with LHL in the context of health care communication. By synthesizing evidence from qualitative and mixed methods studies with qualitative findings, this review will provide a comprehensive overview of how these psychosocial processes influence patient-provider interactions.

The insights generated will contribute to a deeper understanding of the relational dynamics that shape health communication and highlight how shame and concealment may reinforce existing inequities in care. These findings can inform the design of targeted interventions, guide professional education and training for health care providers, and support the development of organizational- and policy-level strategies to foster more equitable communication practices.

Ultimately, this review aims to provide a foundation for future empirical research and practical initiatives that seek to reduce the hidden barriers posed by shame and concealment, thereby promoting more inclusive, patient-centered, and effective health care communication.

## Abbreviations

LHL: low health literacy
MeSH: Medical Subject Headings
PRISMA-ScR: Preferred Reporting Items for Systematic Reviews and Meta-Analyses extension for Scoping Reviews
